# Neural Dynamics of Karaoke-Like Voice Imitation in Singing Performance

**DOI:** 10.3389/fnhum.2020.00135

**Published:** 2020-04-28

**Authors:** Sascha Frühholz, Wiebke Trost, Irina Constantinescu, Didier Grandjean

**Affiliations:** ^1^Department of Psychology, University of Zürich, Zurich, Switzerland; ^2^Neuroscience Center Zurich, University of Zurich and ETH Zurich, Zurich, Switzerland; ^3^Department of Psychology, University of Oslo, Oslo, Norway; ^4^Swiss Center for Affective Sciences, University of Geneva, Geneva, Switzerland; ^5^Department of Neurology, University Hospital Geneva, Geneva, Switzerland; ^6^Department of Psychology, University of Geneva, Geneva, Switzerland

**Keywords:** voice, singing, auditory system, plasticity, neural network

## Abstract

Beyond normal and non-imitative singing, the imitation of the timbre of another singer’s voice, such as in Karaoke singing, involves the demanding reproduction of voice quality features and strongly depends on singing experience and practice. We show that precise voice imitation in a highly proficient and experienced vocal imitator, even in the absence of *external* auditory voice feedback, largely drew on *internal* cortico-subcortical auditory resources to control voicing errors based on imagined voice performance. Compared to the experienced vocal imitator, singers of a control group without experience in voice imitation used only sensorimotor feedback and demanding monitoring resources for imitation in the absence of voice feedback, a neural strategy that led, however, to a significantly poorer vocal performance. Thus, only long-term vocal imitation experience allows for the additional use of internal auditory brain resources, which result from training-induced brain plasticity, and which enable accurate vocal performance even under difficult performance conditions.

## Introduction

Obtaining proficiency in singing and voice imitation requires many years of intensive training. This training often involves learning from and imitation of vocal models, a learning procedure that humans share for example with songbirds (Aronov et al., 2008), and which has its roots in vocal learning (Prat et al., 2015) and infant vocal play (Elowson et al., 1998) across many species. Voice imitation develops with vocal training based on accurate voice feedback processing. This imitation helps novice singers (Dalla Bella and Berkowska, 2009) and amusic individuals (Tremblay-Champoux et al., 2010) to improve their singing performances. Beyond normal and non-imitative singing, the imitation of vocal models involves the reproduction of several vocal acoustic features that contribute to the perceptual quality of the singing voice, such as during Karaoke singing. An individuals’ voice profile, and especially of the singing voice, can be quite unique. An individual voice is characterized by the specific composition of the fundamental frequency originating from the vibration of the vocal folds (i.e., the vocal source signal), and by the vocal formant profile generated by filtering the vocal source signal via the specific anatomy of the vocal tract and cavities, especially the oral cavity. The individual anatomy of the vocal tract imposes limits on the variability of the vocal profile and voice quality of individuals, which defines the vocal register of an individual and which is highly relevant of singing voice imitation.

Voice quality imitation during Karaoke-like singing is thus more demanding than non-imitative singing because voice imitation requires adjustment of certain motoric vocal tract parameters and dynamics to another individuals’ voice profile, which could be very different from personal voice profiles and which might be successful to a variable degree. Specifically, imitating the (singing) voice of another individuals requires one to reproduce certain spectral and temporal voice features, such as vocal pitch, vocal timbre and formants, vocal clearness or roughness as well as timing, temporal (ir-)regularities, and/or articulation, respectively. To control for these spectral and temporal voice features, accurate voice imitation needs to integrate sensorimotor and auditory feedback processing of one’s own performance (Zarate et al., 2010; Zarate, 2013). Both types of feedback processing usually improve with vocal training to support a better vocal performance (Kleber et al., 2010).

**FIGURE 1:**
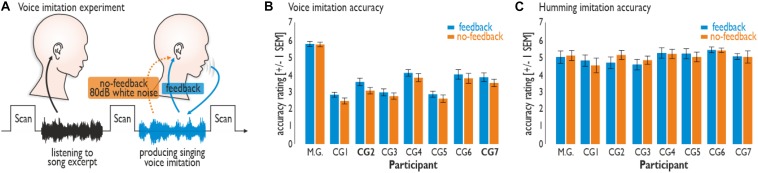
Experimental set-up and vocal performance. **(A)** Participants were asked to passively listen to an 8 s song excerpt in the silent break between brain scans. In the silent break that followed, participants were asked to imitate the singing voice (Experiment 1) or monotonously hum (Experiment 2) according to the song excerpt heard immediately beforehand. Participants either received voice feedback (feedback condition) or did not (no-feedback condition; feedback blocked by 80 dB white noise). **(B)** M.G. revealed a better performance than did all seven participants of the control group (CG1–7) during the voice imitation experiment (Experiment 1). Imitation accuracy was generally lower during the no-feedback compared to the feedback condition. **(C)** Vocal humming performance (Experiment 2) revealed no difference between the feedback and no-feedback condition, nor between the singers.

To specifically test the important role of auditory feedback during the demanding imitation of voice quality, we established an innovative Karaoke-like singing experiment adapted to a brain scanning environment and invited one of the most famous voice imitators to take part in it. This highly trained professional singer and voice imitator (referred to as “M.G.”) is uniquely able to imitate the singing voice of an enormous number of well-known singers. The neural dynamics underlying his voice imitation performances were compared to those of a control group (CG) of singers with singing experience of 8–10 years, but without any in-depth experience in voice imitation. All singers were asked to take part in two different experiments (Figure 1A) while we recorded their brain activity with functional magnetic resonance imaging (fMRI).

The two experiments involved different levels of voice imitation difficulty that were supposed to differentially rely on the level of vocal imitation training. We asked participants to imitate the voice quality of 8-s song excerpts from eight songs of six famous singers, such as Louis Armstrong, Jacques Brel, and Mylène Farmer (Supplementary Table S1). All singers were equally familiar with these songs and reported similar imitation difficulty for them. Experiment 1 required high-demand vocal imitation during the Karaoke-like reproduction of a famous singer’s voice quality. Experiment 2 required only low-demand vocalizations during the simple humming of the same songs as in Experiment 1 without imitation of voice quality and melodic aspects. Both experiments included vocal performance, either with auditory voice feedback or without. For the latter condition, external voice feedback was blocked by 80-dB white noise played back on the singer’s headphones making own voice feedback barely identifiable through bone conduction alone.

## Materials and Methods

### Participants

The experiment included one professional voice imitator (PS) and a CG of non-experienced voice imitators. As professional imitator we invited Michael Gregorio, who is a French male professional singer and voice imitator (referred to as “M.G.”; age 27 years; 11 years of daily vocal training and professional singing experience), to take part in the experiment. M.G.: has a tenor vocal range, and he can imitate the singing voice of a large number of different well-known male and female singers. M.G. was familiar with all songs that were used here to perform vocal imitations.

Additionally, we recruited seven healthy male participants from the University of Geneva as a CG (mean age 27.71 years, SD = 3.86, age range 22–34 years; tenor vocal range). Participants in the CG had an experience of 8–10 years of singing experience in non-professional contexts (e.g., choirs, music bands), and with regular singing practice and performance roughly at a weekly basis. We chose to include these singers with 8–10 years of singing experience to match the approximate amount of years that M.G. was singing on a professional basis at the time of the experiment. All participants were right-handed, had normal or corrected-to-normal vision and had normal hearing abilities. No participant presented a neurologic or psychiatric history. All participants gave informed and written consent for their participation in accordance with ethical and data security guidelines of the University of Geneva. The participants of the CG were monetarily reimbursed for their participation in the experiment. The study was approved by the local ethics committee of the University of Geneva.

### Stimuli and Experimental Procedure

#### Voice Imitation Experiment (Experiment 1)

The main experiment included two experimental blocks. These blocks were split in two parts, where in one part participants were singing while listening to their own vocal productions (feedback), while in the other part they were singing while their own vocal feedback was completely blocked by presenting white noise at 80 dB SPL over fMRI compatible headphones (no-feedback). In total there were 32 trials for each condition of singing with feedback and singing without feedback. The experiment also included eight silent trials per blocks, for which no song was presented, and no singing was required. Silent trials were included to enable a better estimate of baseline neural activity during the first level modeling of the brain data.

A single trial consisted of two parts. In the first part, participants passively listened to an 8 s-excerpt from a famous song (Supplementary Table S1) that was presented in the silent gap between image acquisitions, while in the second part participants were asked to imitate the singing voice that they heard immediately before. No background music was played during the active singing phase. Excerpts were extracted from famous songs that were in the repertoire of M.G. in the year 2011 and for which we could extract at least 8 s of continuous singing. The 8 s excerpts were randomly selected, and none of the singers were involved in the selection of the excerpts. Participants were specifically asked to imitate the voice quality as heard in the song. Participants were asked to sing during the next silent gap during image acquisition following the silent gap for the song presentation. For this active singing task, participants were instructed to specifically focus on imitating the voice quality of the singer from the song excerpt, with a priority on voice quality and timbre features as well as temporal singing style and articulation features. To familiarize participants with the songs and the voice quality of the singers, all songs were given to participants one day before the experiment, but participants did not know which 8 s segments from the songs were going to be presented during the experiment. In total we presented 16 excerpts from 8 famous French or American songs from a total of 6 different singers. Thus, we extracted two different 8 s excerpts from every song. The excerpts were normalized to have a mean intensity of 70 dB SPL. Each participant was given a 20 min practice session before the fMRI experiment.

#### Humming Experiment (Experiment 2)

Similar to the experiment setup for the singing experiment, we also included another experiment consisting of vocal humming. This humming experiment consisted of one block and was again split into one part with feedback and one part without feedback. During this experiment we asked participants to all only croon/hum the song excerpts that they heard immediately before with only minor pitch variations. Song excerpts were the same as in the main experiment. This humming experiment involved vocal production related to the original songs, but without much effort of vocal modulation to reproduce the vocal melody of the song and without imitation of vocal features of the original voice. In total there were again 32 trials for each condition of humming with feedback and of humming without feedback.

#### Voice Localizer Experiment

We used 8s sound clips taken from an existing database (Belin et al., 2000) to identify regions in the bilateral superior temporal cortex that are generally sensitive to human voices in each group of patients. To this end, we used sound clips representing 20 sequences of human voices and 20 sequences of animal or environmental sounds. Each sound clip was presented once at 70 dB SPL. The scanning sequence also contained 20 of 8 s silent events. The participant had to passively listen to these stimuli.

#### White Noise Localizer Experiment

During the white noise localizer scan, we presented 30 trials of uniform white noise (1 Hz–8 kHz) at an intensity level of 80 dB SPL and a 1500 ms duration as well as 30 silent trials. Stimuli were presented in the silent gap between image acquisitions. The white noise localizer scan was included to reveal neural activity resulting from presented white noise in the main experiment use to mask vocal feedback during singing.

#### Postexperimental Rating

After the experiment, each participant was asked to evaluate each song excerpt that they listened to during the main experiment on an 8-point scale according to two-dimensions: (a) how familiar they were with the song and (b) how difficult it was to interpret and imitate the excerpt.

#### Acoustic Analysis and Perceptual Evaluation of Singing Performance

From each vocal performance of each participant we extracted the level of acoustic intensity of singing in terms of dB SPL. We found a significant, but marginal difference in terms of the mean (*X*^2^ = 4.50, *df* = 1, *p* = 0.034; Friedman test with participants as replication factor) of voice imitation during the feedback (M_INT_ = 66.27 dB) compared to the no-feedback condition (M_INT_ = 67.83 dB) for the whole sample of participants, but there was only a tendency between participants (*X*^2^ = 13.83, *df* = 7, *p* = 0.054; Friedman test with feedback/no feedback as replication factor). All statistical test were performed using Matlab’s statistics toolbox (version 11.6).

Furthermore, we performed, first, a perceptual evaluation of the singing performance by seven independent raters (three females, mean age 30.86 years, SD = 6.08, age range 23–43 years) who did not take part in the main experiments and who were unfamiliar with the experience of the vocal imitator. These raters were recruited by University and public announcements (i.e., on social media), and included both University members and persons from the general public. Raters were asked to assess the quality and level of vocal imitation for each 8 s singing recording block by a perceptual comparison to the original song excerpt in a direct comparison. Raters were instructed to mainly focus on the vocal timbre and voice quality features, but also on temporal singing features. Ratings were performed using a 7-point Likert scale ranging from “1” (very inaccurate imitation) to “7” (very accurate imitation), including “4” (medium accurate imitation) as the midpoint of the scale. Second, we also performed a perceptual evaluation of the humming performance during the humming experiment by another seven independent raters (four females, mean age 26.44 years, SD = 3.98, age range 21–35 years) who did not take part in the main experiment. The rating was done in the same way as for the singing performance, but raters were asked to mainly focus on the accurateness of temporal pitch and timing features.

### Image Acquisition

Functional imaging data for the main experiment were recorded on a 3T Siemens Trio System (Siemens, Erlangen, Germany) using a T2^∗^-weighted gradient echo planar imaging sequence (TR = 12.48 s, TA = 3.12 s, TE = 30 ms, FA = 90°, 50 slices, voxel resolution 2 mm^3^, distance factor = 20%). We used a sparse temporal acquisition protocol for the main experiment, which allowed presentation of auditory stimuli in the silent gap between volume acquisitions. It also allowed us to record the singing performance of the participants (see below), which were thus unaffected by the background scanner noise. Functional imaging data of the white noise localizer scan were recorded using a fast-sparse temporal sampling protocol (TR = 3.30 s, TA = 1.60 s, TE = 30 ms, FA = 90°, 25 slices, voxel resolution 2 mm^3^, distance factor = 20%) and a partial volume acquisition covering the whole auditory cortex (AC). Functional data for the voice localizer scan were recorded using a continuous acquisition (TR = 2.1 s, TE = 30 ms, FA = 80°, 36 slices, voxel resolution 3.2 mm^3^, distance factor = 20%). Finally, a high-resolution magnetization prepared rapid acquisition gradient echo T1-weighted sequence (1 mm slices, TR = 1900 ms, TE = 2.27 ms, TI = 900 ms, FoV = 296 mm, in-plane 1 × 1 mm) was obtained in sagittal orientation to obtain structural brain images from each subject.

### Image Analysis

We used the statistical parametric mapping software SPM (Version 12; Welcome Department of Cognitive Neurology, London, United Kingdom) for preprocessing and statistical analysis of functional images from the main and from the control experiment. Functional images were realigned and co-registered to the anatomical image. We used the New Segment option in SPM (Ashburner and Friston, 2005) to segment participants’ anatomical brain scans into white matter and gray matter parts based on a probabilistic approach by comparing participants’ brains to standard probabilistic brain tissue classes. To normalize brain images to the Montreal Neurological Institute (MNI) space we then subjected all participants’ gray and white matter images to a unified segmentation and group-wise normalization approach with the DARTEL toolbox (Ashburner, 2007). DARTEL uses a group-wise registration of individual gray and white matter tissue segments to their iteratively evolving group average that is finally registered to the MNI template space. During this process, DARTEL estimates individual flow fields for normalization of each individual brain to the MNI space. Functional images were resampled to a voxel size of 2 mm^3^ and spatially smoothed by using an isotropic Gaussian kernel of 6 mm^3^ full-width at half-maximum.

For the group-level analysis, including comparison between the data of a single participant (M.G.) to the data of a group of participants (CG), there are three possible approaches that all have advantages and disadvantages. One approach uses an “artificial” two-sample *t*-test approach (Roswandowitz et al., 2017) with the limitation that the variance in one group cannot be estimated given only one participant in one group. For this case the variance of the single participant (M.G.) is set equal to the estimated variance in the CG. This however is a very unlikely assumption especially when comparing an experienced vocal imitator with non-experienced imitators. Second, single contrast of activity in M.G. compared separately to each participant of the CG can be entered into a group-level one-sample *t*-test (Elforgani et al., 2014), which however most likely overestimates the signal difference between M.G. and the members of the CG. Third, all data from each participant can be entered in a group-level fixed-effect (FFX) modeling approach (Xu et al., 2015), which models all data in a single general linear model (GLM), with the disadvantage of missing the participant or group factor as random effect factor. The latter option however allows for a more direct investigation of interaction contrasts between conditions in a single model, and most likely gives more valid estimations of the signal differences between M.G. and the CG. In the case of the present study, we found that the latter approach revealed a consistent and balanced estimation of brain activity in gray matter across several contrasts performed without over- or under-estimating group differences. This approach also led to less spurious activation in unlikely brain regions, such as the ventricles or white matter, as found with the other two analysis approaches. As this single GLM approach has been used previously, and led to a consistent estimation of group differences in brain activity, we here opted for taking a FFX analysis approach to the brain data.

We thus used a single general linear FFX model for the statistical analyses of functional data including data from both the main and the control experiment as well as data from both M.G. and the data of the CG. This approach allowed us to model within and across subject data variance in a single statistical model, and to directly compare functional brain activity in M.G. compared with activity of the CG. Experimental events within and across participants were defined by boxcar functions defined by the onset and duration of the auditory stimuli as well as by the onset and the duration of singing. These boxcar functions were convolved with a canonical hemodynamic response function. Separate regressors were created for each experimental condition. Six motion correction parameters for each participant were also included as regressors of no interest to minimize false positive activations that were due to task-correlated motion.

For the main experiment we entered five conditions per participant in the GLM, that is, singing with and without feedback, humming with and without feedback, and one regressor that modeled every occurrence of a song excerpt prior to every singing part. We computed contrasts especially between the conditions of no-feedback and the conditions with feedback for both the singing and for the humming experiment. The resulting activation was compared between PS and CG using an interaction contrast. The resulting statistical maps were thresholded at a combined voxel threshold of *p* < 0.005 (uncorrected) and a minimum cluster extent of *k* = 56, which corresponds to *p* < 0.05 corrected at the cluster level as determined using the 3DClustSim algorithm implemented in the AFNI software^1^ [version AFNI_18.3.01; including the new (spatial) autocorrelation function (ACF) extension] according to the estimated smoothness of the data across all contrasts.

For the voice localizer scan, we contrasted vocal against non-vocal animal and environmental stimuli across all participants. Contrast images were threshold of *p* < 0.005 (uncorrected) and a cluster extent of *k* = 93 voxels corresponding to *p* < 0.05 corrected at the cluster level. We determined voice-sensitive regions along the STG and STS in both hemispheres. For the white noise localizer scan we contrasted the presentation of white noise stimuli against baseline. The resulting statistical maps were thresholded with a combined voxel and cluster threshold of *p* < 0.005 (uncorrected) and a minimum cluster extent of *k* = 81 corresponding to *p* < 0.05 corrected at the cluster level.

### Functional Connectivity Analysis

To determine the functional connectivity for regions that we identified as showing higher activity during the no-feedback relative to the feedback condition in M.G. and the CG during the singing experiment, we also performed a functional connectivity analysis using these regions as seed regions in a psycho-physiological interaction (PPI) analysis. The PPI analysis was set up as a GLM for each of the seed regions including three regressors. As a first regressor we included the extracted and deconvolved time course of functional activity in a seed region (the physiological variable). The second regressor included the comparison between angry and neutral voices during the explicit task (the psychological variable). Specifically, we created a time course regressor for the task including as many sampling points as for the physiological variable. The values in this regressor were set to “1” for trials including the no-feedback condition and to “−1” for trials including the feedback condition. The third regressor, finally, included the interaction between the first two regressors. This interaction was created by a point-by-point multiplication of the time course for the physiological variable and the time course for the psychological variable. The last regressor was the only regressor of interest, whereas the psychological variable and the deconvolved time course served as regressors of no interest in each PPI analysis. The inclusion of the first two regressors ensures that the resulting functional activation is solely determined by the *interaction* between the physiological variable and the psychological variable. These data for the explicit and implicit task were separately entered into a (PPI) analysis for each of the five seed regions.

Similar to the main experiment, this analysis was again setup as a single general linear FFX model for each seed region including all three regressors of the PPI analysis for each participant, thus resulting in a model including 24 regressors. We computed contrasts for activity in M.G. relative to the CG, or vice versa. The resulting statistical maps were thresholded at a combined voxel threshold of *p* < 0.005 (uncorrected) and a minimum cluster extent of *k* = 56, which corresponds to *p* < 0.05 corrected at the cluster level (see above). This cluster extend threshold was the minimum necessary threshold that we determined according to the estimated smoothness of each PPI analysis.

For the voice imitation experiment (Experiment 1) we performed this analysis on seven seed regions that showed significant activity either for M.G. (left aIFG, left aSTG, left pSTG, left IPS, right Cd) or for the CG (bilateral pIFG, left SOC, right Cd). We have to note that activity in a region can be differentially predictive for functional connectivity even when its activity is lower in one group. The following peak voxels were used as seed regions based on a 3 mm sphere around these peak voxels: left aIFG (MNI*xyz* −42 34 −16), left (−56 18 22) and right pIFG (62 12 22), left aSTG (−48 14 −18), left pSTG (−56 −4 0), left SoC (−42 −30 24), right Cd [6 2 8 and (8 20 10)], and left IPS (−28 −74 54).

Functional connectivity during the humming experiment (Experiment 2) included the same major seed or target brain areas as for the singing experiment, but with additional areas in medial frontal cortex (MFC) and the limbic system. For the humming experiment we performed the functional connectivity analysis on eight seed regions that showed significant activity either for M.G. (left MFC and SFG) or for the CG (bilateral MC, left MFC, left IPS, right aINS, and right pIFG). The following peak voxels were used to create seed regions: left MFC [(−14 50 20) and (−12 60 0)], left SFG (−18 6 50), right pIFG (62 10 22), left (−62 −4 28) and right MC (64 −6 28), right aINS (36 −2 14), and left IPS (−28 −74 24). These seed regions were used to determine the functional connectivity for both M.G. and the CG. We have to note that though these regions were active only in M.G. or the CG, the use of these seed region for both M.G. and the CG is justified because a seed region can show significant functional connectivity even if it was less active in M.G. or in the CG. Less activity can still mean that a region showed significant activity above baseline.

We finally also quantified the neural network architecture for the voice imitation and the humming experiment separately for M.G. and the CG. We defined major brain areas for the singing experiment: lateral frontal areas included the IFG and the dorsolateral prefrontal (dlPFC, consisting of middle and superior frontal gyrus); motor areas included motor cortex (MC), somato-sensory cortex (SoC), and the caudate nucleus [Cd, as part of fronto-striatal motor loops (Alexander, 1986)]; parietal areas represented by the intra-parietal sulcus (IPS); auditory areas consisted of voice-sensitive the AC [composed of primary AC, planum polare (PPo), and planum temporal (PTe)], the STG, and the STS; and the cerebellum. For the humming experiment, we used the same major brain areas but also used the following areas: MFC, the anterior insula (aINS) as additional part of the lateral frontal areas; and the limbic system composed of the amygdala and the hippocampus. For each of these major brain areas we quantified the absolute and relative number of seed and target connections. The relative number was determined by the amount of absolute connections for each brain area divided by the number of total connections for M.G. or the CG.

## Results

### Accuracy of Vocal Performance

We were interested first in the accuracy of the vocal performances in the absence of voice feedback, which is usually central to a convincing performance. Vocal performance accuracy was assessed through perceptual evaluations by seven independent raters (see section “Materials and Methods”). Voice imitation performance accuracy (Experiment 1) was higher overall for M.G. than for the CG and higher for the feedback than for the no-feedback condition across all singers (Figure 1B). M.G. revealed an overall better performance than did the participants of the CG (CG1–7) during the voice imitation experiment (Friedman test: χ^2^ = 71.36, *df* = 7, *p* < 0.001). M.G. also achieved higher scores in imitation accuracy compared to each singer of the CG (Wilcoxon signed rank tests: all *Z*s > 3.30, all *p*s < 0.001). Imitation accuracy was generally lower during the no-feedback compared to the feedback condition (Friedman test: χ^2^ = 5.77, *df* = 1, *p* = 0.0163), especially for two singers from the CG (CG2 and CG7; Wilcoxon signed rank tests: all *Z*s > 2.03, all *p*s < 0.042).

No such differences across conditions or singers were found for the low-demand humming experiment (Experiment 2) (Figure 1C). Vocal humming performance accuracy revealed no difference between the feedback and no-feedback condition (Friedman test: χ^2^ = 0.36, *df* = 1, *p* = 0.551), nor between the singers (Friedman test: χ^2^ = 10.50, *df* = 7, *p* = 0.162). Thus, the higher level of vocal imitation training in M.G. resulted in a much better voice imitation performance for imitation accuracy compared to the CG during demanding voice imitation, but not during vocal humming.

### Postexperimental Ratings

M.G. (FAM = 5.36) tended to be more familiar with song excerpts compared to the CG (all FAM < 3.50), but there was no significant difference in the ratings across the whole sample for all songs (Friedman test with the factors *participant* and *song*: χ^2^ = 13.01, *df* = 7, *p* = 0.072). There was also no significant difference between the ratings of the difficulty of the vocal imitations (M.G.: DIFF = 4.59; CG: all DIFF < 3.94; Friedman test: χ^2^ = 5.96, *df* = 7, *p* = 0.544). The difficulty (Spearman rank correlations; feedback condition: all abs(*r*’s) < 0.63, all *p*’s > 0.106; no-feedback condition: all abs(*r*’s) < 0.651, all *p*’s > 0.089) and the familiarity ratings (Spearman rank correlations; feedback condition: all abs(*r*’s) < 0.45, all *p*’s > 0.273; no-feedback condition: all abs(*r*’s) < 0.729, all *p*’s > 0.050) were not correlated with the imitation accuracy ratings across all songs.

### Functional Localizer Experiments

**FIGURE 2:**
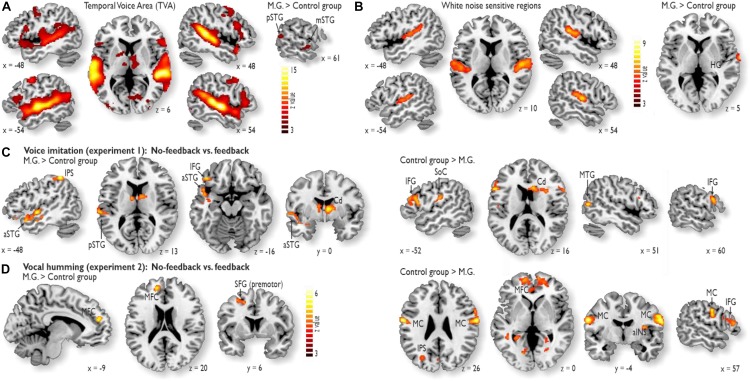
Functional activity during the localizer experiments and during the voice and humming experiment. **(A)** The voice localizer experiment revealed extended activity in the bilateral auditory cortex (AC), especially in the STG. M.G. showed higher voice sensitivity in the right posterior STG (pSTG) and the mid STG (mSTG) compared to the control group (right panel). **(B)** The white noise localizer experiment revealed noise sensitivity in the bilateral primary AC and posterior secondary AC. M.G. showed higher white noise sensitivity in right Heschl’s gyrus (HG) (right panel). **(C)** While voice feedback was blocked during voice imitation (Experiment 1), activity in M.G. (left panel) was increased relative to that in the control group in the left pSTG and anterior STG (aSTG), left inferior frontal gyrus (IFG), left intra-parietal sulcus (IPS) and right caudate nucleus (Cd). For the same contrast, activity was increased in the bilateral IFG, right Cd, left somato-sensory cortex (SoC) and right middle temporal gyrus (MTG) for the control group relative to M.G. The latter MTG activity was located outside the TVA. **(D)** While vocal feedback was blocked during the humming experiment (Experiment 2), M.G. showed higher activity relative to the control group in the left medial frontal cortex (MFC) and dorsolateral prefrontal cortex (dlPFC; i.e., in superior frontal gyrus, SFG), whereas the control group relative to M.G. showed higher activity in the bilateral motor cortex (MC), MFC, anterior insula (aINS), IPS, and right IFG. All brain activations are thresholded at *p* < 0.05 corrected at the cluster level.

We performed two separate functional localizer experiments in addition to the main fMRI experiments. First, we performed a voice localizer experiment to determine voice-sensitive regions in AC (Belin et al., 2000). This experiment revealed extended activity in the bilateral AC, especially in the superior temporal gyrus (STG). This area is referred to as the temporal voice area (TVA) (Figure 2A). M.G. showed higher voice sensitivity in the right posterior STG [pSTG; (MNI *xyz* 66 −12 −8), *z* = 5.24] and the mid STG [mSTG; (66 −52 14), *z* = 3.52] compared to the CG (right panel). This right pSTG/mSTG activity did not overlap with activations in the main experiments (see below; Figures 2C,D).

Second, we performed a white noise localizer experiment to determine white-noise sensitive regions, which were sensitive to the white noise that was used to block own-voice auditory feedback. This experiment revealed noise sensitivity in the bilateral primary AC and posterior secondary AC (Figure 2B). M.G. showed higher white noise sensitivity in right Heschl’s gyrus [HG; (68 −4 0), *z* = 6.15] (right panel). This right HG activity did not overlap with activations in the main experiments (see below; Figures 2C,D).

### Functional Activations During Voice Imitation and Vocal Humming

During Experiment 1 for voice imitation in the absence of voice feedback relative to the presence of voice feedback, M.G. revealed *common* as well as *distinct* neural patterns of activity and connectivity compared to the CG. In terms of common activity, both M.G. and the CG revealed common activity in the Cd of the basal ganglia and the left anterior inferior frontal gyrus (aIFG), with additional activity in the right posterior IFG (pIFG) for the CG (Figures 2C,D and Supplementary Table S2).

In terms of distinct activity, a striking finding was that M.G. specifically revealed enhanced activity in the left voice-sensitive AC (Figure 2A; see sections “Materials and Methods” and “Results”). No auditory cortical activity was found in the CG. This auditory cortical activity in M.G. was located in anterior (aSTG) and pSTG and thus outside auditory cortical regions that were sensitive to simple white noise used to block the voice feedback (Figure 2B). In addition to the activity in the auditory regions (Figure 2C), M.G. also revealed enhanced activity in the left intra-parietal sulcus (IPS) during imitation without feedback (Supplementary Table S2A). Compared to M.G., who had higher activity in auditory regions and the IPS, the CG revealed increased activity in the inferior SoC for voice imitation without feedback (Figure 2C and Supplementary Table S2B). This activity was located in the areas that represent sensory input from the mouth region and the vocal tract.

Activity during the humming experiment (Experiment 2) figured as a low-demand vocal reference task. Humming in the absence of feedback revealed no activity in auditory regions in both groups, but activity in the MFC and the premotor cortex located on the superior frontal gyrus (SFG) for M.G. (Figure 2D and Supplementary Table S3A), as well as in the MFC, MC, aINS, and right pIFG for the CG (Figure 2D and Supplementary Table S3B).

### Functional Connectivity Analysis

**FIGURE 3:**
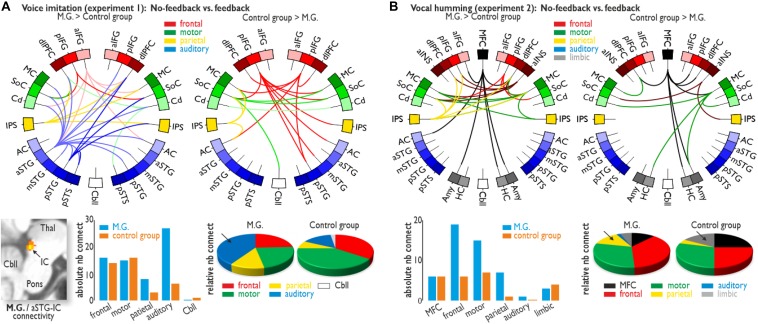
Functional brain connectivity. **(A)** Functional connectivity for the voice imitation experiment (Experiment 1). While voice feedback was blocked, M.G. showed widespread functional connectivity between frontal (IFG and dlPFC), sensorimotor (MC, SoC, and Cd), parietal (IPS) and auditory regions [AC, aSTG/mSTG/pSTG, and posterior superior temporal sulcus (pSTS)] (upper left panel), as well as additional connectivity between the aSTG and subcortical IC (lower left panel). For the same contrast, functional connectivity was found between only the frontal, sensori-motor, parietal, and Cbll areas for the control group (upper right panel). Although for all major target regions, the absolute number of connections was largely comparable between M.G. and the control group (lower middle panel), M.G. showed a dense connectivity to auditory regions during the no-feedback condition, which was also confirmed by quantification of the relative number of auditory connections (arrow in lower right panel). **(B)** Functional connectivity for the humming experiment (Experiment 2). We found functional connectivity especially between the MFC and lateral frontal (IFG, dlPFC, aINS) areas for both M.G. and the control group. Whereas M.G. showed an absolute increase in frontal, motor and parietal connections, the relative proportion of parietal connections was larger in M.G. (arrow in lower right panel), and the relative proportion of limbic connections [amygdala (Amy) and hippocampus (HC)] was larger in the control group (arrow). All functional activations and connections are thresholded at *p* < 0.05 corrected at the cluster level.

The importance of auditory cortical activity during voice imitation in the absence of feedback in M.G. compared to the CG is strengthened by an additional brain connectivity analysis that revealed collaboration and information exchange between brain areas. To asses the functional connections for the no-feedback relative to the feedback condition in the voice imitation experiment (Experiment 1), we performed a PPI analysis on seven seed regions that showed significantly higher activity either for M.G. (left aIFG, left aSTG, left pSTG, left IPS, right Cd) or for the CG (bilateral pIFG, left SoC, right Cd) (Figure 3A). These seed regions and corresponding target regions were grouped into five major brain parts, represented by lateral frontal, motor, parietal, auditory, and cerebellar areas. These major areas are representative of the voice production and voice processing network (Frühholz et al., 2014a, 2016; Klaas et al., 2015). For each of these major brain areas we quantified the absolute and relative number of seed and target connections. The relative number was determined by the amount of absolute connection for each brain area divided by the number of total connections for M.G. or the CG.

M.G. revealed dense functional connectivity of auditory regions with all other major brain areas compared to the CG (Figure 3A and Supplementary Table S4). M.G. also revealed connectivity between a left cortical auditory region in the aSTG and a subcortical auditory node of the ascending auditory pathway, namely the inferior colliculi (IC). Contrary to the importance of neural auditory system in M.G. during voice imitation with no feedback, the CG demonstrated a strong reliance on the sensorimotor system. This higher sensorimotor effort in less trained singers was also indicated by the relative number of functional connections (i.e., relative to the total number of connections) involving motor regions in the CG, accompanied by connectivity to the cerebellum (Figure 3A and Supplementary Table S4).

Functional connectivity for the no-feedback relative to the feedback condition in the humming experiment (Experiment 2) was performed as a PPI analysis on eight seed regions that showed significant activity either for M.G. (left MFC and SFG) or for the CG (bilateral MC, left MFC, left IPS, right aINS, and right pIFG). Although both groups showed similarities in their functional connections during vocal humming in the absence of feedback, especially connections of MFC and lateral frontal [IFG, aINS, and dorsolateral prefrontal cortex (dlPFC)] areas, the number of functional connections, in terms of relative numbers, was slightly increased in M.G. This included the IPS again “forwardly” interfacing to the MC and dlPFC, whereas in the CG, we found slightly increased connectivity of the MFC and of motor areas to limbic regions (Figure 3B and Supplementary Table S5).

## Discussion

The neural strategy of solving the demanding task of voice imitation in the absence of voice feedback seems largely influenced by the level of vocal imitation training. On the most general level, we revealed a fundamental difference in terms of neural mechanisms between the experienced vocal imitator and the CG. While frontal control and external sensorimotor resources were mainly used by the less trained singers of the CG to solve this task of voice imitation without feedback from the own voice, mainly cortico-subcortical auditory regions were used by the experienced vocal imitator as central nodes in a large-scale neural network. The latter neural strategy of using “internal” auditory resources led to better vocal imitation performance during the difficult performance condition of imitation in the absence of feedback, and thus might be one of the specific neural mechanism resulting from intense vocal imitation training.

Although the experienced vocal imitator and the CG showed fundamental neural differences, which will be discussed in more detail below, there were also some common neural resources that were used by both groups. For example, we found common neural activity in the basal ganglia and the IFG. These regions usually support dynamic voice quality management (Frühholz et al., 2014a) and monitoring (Frühholz et al., 2014b). An important consideration is that these regions represent neural nodes that have so far been neglected in neural network models of singing (Zarate, 2013) and thus might underlie the more demanding task of voice quality imitation in general. The additional right IFG activity in the CG might indicate their higher effort in voice monitoring of their performance, which was also indicated by a less accurate vocal performance compared to the experienced vocal imitator.

In addition to these instances of common neural activity, we found several important differences in the neural mechanisms for singing without own-voice feedback. Concerning distinct neural mechanism resulting from voice imitation training, we found auditory cortical for M.G., but not for the CG. The cortical activity was located in the voice-sensitive cortex (Frühholz and Grandjean, 2013), and was surprising to find, given that it was found in the absence of voice feedback. Thus, this activation might not indicate an “external” voice feedback processing, but rather the “internal” imagination of vocal feedback. Imagination of auditory sensory stimulation enhances activity in the AC (Kraemer et al., 2005), especially in higher-level AC (Kosslyn et al., 2001) as found in the present study. The imagination of auditory voice feedback in M.G. might considerably improve the accuracy of vocal performance even in the absence of external feedback.

For M.G. we also found distinct activity in the parietal cortex, especially in the IPS. The IPS is usually supposed to be a bidirectional interface between auditory regions registering external vocal signals and motor regions that program vocal output (Andersen and Buneo, 2002; Zarate, 2013). This interfacing role of the IPS, in the absence of external voice feedback, might be used by frontal regions as an attempt to access any potentially available information in the AC, which might be a primary neural strategy in experienced vocal imitators. This supposed “backward” interfacing role of the IPS was found in the left brain of the CG, but only in terms of functional connectivity (i.e., in the absence of primary IPS activity), whereas the IPS showed a different connectivity in M.G., mainly “forwardly” interfacing to the MC and dlPFC. This finding might support the selection of relevant motor action programs based on elaborate cognitive plans for vocal movements (Andersen and Buneo, 2002), and these elaborate plans might result from the higher level of vocal imitation training in M.G.

As mentioned above, less trained singers of the CG solve this problem of voice imitation without feedback by mainly relying on sensorimotor resources. We accordingly found distinct activity in the SoC for the CG. Activity in SoC is usually assumed to represent the level of vocal training (Kleber et al., 2013), which would predict higher activation in the SoC for M.G. However, in the absence of auditory own voice feedback, the functional role of the SoC might be different, such as showing higher activity in less trained singers, and thus reflecting the higher sensorimotor effort undertaken by singers of the CG rather than the level of training in professional singers. This SoC activity in the CG was also accompanied by connectivity of the SoC to the cerebellum that usually supports fine-motor vocal adjustments (Frühholz et al., 2014b, 2016).This overall neural strategy of relying on sensorimotor information, however, led to less accurate performance in the CG.

Unlike during voice imitation, the vocal performance during low demanding vocal humming was comparable between the experienced vocal imitator and the CG. During vocal humming, the CG revealed a distinct activity that was comparable to their neural activity during voice imitation, and was located in IFG. This IFG activity was located close to the posterior IFG activity during voice imitation, indicating some similarities in the monitoring effort for both vocal tasks in the CG. Besides the IFG, many of these regions found during vocal humming, such as MFC, motor, and premotor cortex, are (in-)directly involved in vocal motor output planning, execution and monitoring (Jürgens, 2002; Zarate, 2013), which was also confirmed by the extensive functional connections of these regions to lateral frontal and sensorimotor areas, including the Cd that temporally sequences and programs vocal motor output (Kotz and Schwartze, 2010; Trost et al., 2014).

Additional to these common and distinct patterns of neural activity during the voice imitation and the vocal humming task, we also determined the functional neural connectivity underlying the vocal performance in these tasks in the absence of voice feedback. During voice imitation in the absence of feedback, we found comparable functional connectivity for the experienced vocal imitator and the CG between frontal, motor, and parietal regions. However, a major difference concerned functional connections involving auditory regions. For the experienced vocal imitator only, we found dense functional connectivity with auditory regions, highlighting the general importance of neural auditory resources for voice imitation in experienced vocal imitators. Next to widespread functional connectivity of auditory regions to other cortical regions in the experienced vocal imitator, we also found a very specific connection of the AC to the subcortical IC. This functional IC connection might specifically indicate the registration of vocalization motor errors at an early and low level of motor and (imagined) voice feedback integration, which happens in the IC (Gruters and Groh, 2012). This low-level integration might lead to finer tuning of a vocal performance and to better voice imitation accuracy.

Similar to the voice imitation task, we found a fronto-motor functional network during the vocal humming task that reflects the motor planning, execution, and monitoring part of vocalizations. However, we also found additional connectivity of MFC and motor areas to limbic areas in both groups that were not found during voice imitation. Concerning this limbic system connectivity, the MFC-amygdala connectivity might be part of a neural network for initiating and emotionally motivating vocalizations (Jürgens, 2002), which seems more important for the less “natural” vocalizations of monotonous humming. The MFC-hippocampus connectivity might be involved in the encoding and retrieval of temporal song sequences (Allen et al., 2016), which is more important for monotonous vocal humming than for melodic voice imitation. No functional connections involving auditory areas were found during vocal humming, except for one MFC-AC connection, which we found for M.G. Thus, vocal humming does not rely much on auditory resources.

It is worthwhile to comment on possible limitations regarding the present study. First, we only investigated one experienced vocal imitator and compared his vocal performance and his brain activity with those of a sample of singers in the CG. However, M.G. is very specific and unique in his ability to imitate other singers’ voices, making it extremely difficult to find additional voice imitators with comparable levels of expertise. We however used sensitive analysis methods to reliably determine performance and neural differences between M.G. and the CG. Second, the study included song excerpts that were mainly taken from the standard repertoire of M.G., which could introduce a bias given that M.G. was practicing and performing these songs more often than the singers of the CG. However, we chose songs and song excerpts that are very familiar to many individuals, and would not need much training for an accurate repetition, but more for an accurate voice imitation. Third, we aimed at including singers in the CG that matched the singing experience of M.G. as closely as possible. We recruited singers with a close vocal register and with approximately the same number of years of singing experience, with the exception that M.G. in total performs many more hours of active singing compared to the CG. But this large amount of hours of active singing is the basis for his high level of ability as a voice imitator. Additionally, while including another CG of singers with a comparable amount of singing hours would have been possible, it likely would have caused problems for matching other features (such as vocal register, vocal training and education, performance activities, etc.). Finally, we also note that in the humming experiment we did not find considerable vocal performance differences as well as neural differences in central brain areas that were of primary interest in the main experiment between M.G. and the CG. The humming experiment was introduced to assess the baseline vocal performance and neural imitation effects in M.G. and the CG.

## Conclusion

Summarizing our data, cortico-subcortical auditory regions were used by an experienced vocal imitator as central nodes in a large-scale neural network during singing voice imitation under a difficult performance condition. The use of these neural auditory resources in the absence of voice feedback, thus in the absence of external auditory input from one’s own voice, points to the use of internal auditory resources probably based on voice imagination. These internal resources might develop with extensive vocal training and might be the neural basis of intense vocal imitation training. This conducive and beneficially neural strategy and neural repertoire of a singing and experienced vocal imitator might thus point to a valid training strategy for facilitating neural and vocal plasticity for singing novices and semi-professional singers.

## Data Availability Statement

All datasets generated for this study are included in the article/Supplementary Material.

## Ethics Statement

The studies involving human participants were reviewed and approved by the Cantonal ethics committee, Zurich. The patients/participants provided their written informed consent to participate in this study. Written informed consent was obtained from the individual(s) for the publication of any potentially identifiable images or data included in this article.

## Author Contributions

SF, IC, and DG designed the study. SF and WT collected and analyzed the data. SF and WT wrote the manuscript. All authors discussed and commented on the final manuscript.

## Conflict of Interest

The authors declare that the research was conducted in the absence of any commercial or financial relationships that could be construed as a potential conflict of interest.

## Footnotes

1 http://afni.nimh.nih.gov/afni

## References

[B1] Alexander G. E. (1986). Segregated circuits linking basal ganglia and cortex. *Annu. Rev. Neurosci.* 9 357–381. 10.1146/annurev.neuro.9.1.357 3085570

[B2] Allen T. A. Salz D. M. McKenzie S. Fortin N. J. (2016). Nonspatial sequence coding in CA1 neurons. *J. Neurosci.* 36 1547–1563. 10.1523/JNEUROSCI.2874-15.2016 26843637 PMC4737769

[B3] Andersen R. A. Buneo C. A. (2002). Intentional maps in posterior parietal cortex. *Annu. Rev. Neurosci.* 25 189–220. 10.1146/annurev.neuro.25.112701.142922 12052908

[B4] Aronov D. Andalman A. S. Fee M. S. (2008). A specialized forebrain circuit for vocal babbling in the juvenile songbird. *Science* 320 630–634. 10.1126/science.1155140 18451295

[B5] Ashburner J. (2007). A fast diffeomorphic image registration algorithm. *Neuroimage* 38 95–113. 10.1016/j.neuroimage.2007.07.007 17761438

[B6] Ashburner J. Friston K. J. (2005). Unified segmentation. *Neuroimage* 26 839–851. 10.1016/j.neuroimage.2005.02.018 15955494

[B7] Belin P. Zatorre R. J. Lafaille P. Ahad P. Pike B. (2000). Voice-selective areas in human auditory cortex. *Nature* 403 309–312. 10.1038/35002078 10659849

[B8] Dalla Bella S. Berkowska M. (2009). Singing proficiency in the majority: normality and “phenotypes” of poor singing. *Ann. N. Y. Acad. Sci.* 1169 99–107. 10.1111/j.1749-6632.2009.04558.x 19673762

[B9] Elforgani M. S. A. Alnawawi A. Rahmat I. B. (2014). The association between clients’ qualities and design team attributes of building projects. *ARPN J. Eng. Appl. Sci.* 9 160–172. 10.1073/pnas.1422679112

[B10] Elowson A. M. Snowdon C. T. Lazaro-Perea C. (1998). “Babbling” and social context in infant monkeys: parallels to human infants. *Trends Cogn. Sci.* 2 31–37. 10.1016/S1364-6613(97)01115-1117 21244960

[B11] Frühholz S. Grandjean D. (2013). Multiple subregions in superior temporal cortex are differentially sensitive to vocal expressions: a quantitative meta-analysis. *Neurosci. Biobehav. Rev.* 37 24–35. 10.1016/j.neubiorev.2012.11.002 23153796

[B12] Frühholz S. Klaas H. S. Patel S. Grandjean D. Fruhholz S. Klaas H. S. (2014a). Talking in fury: the cortico-subcortical network underlying angry vocalizations. *Cereb. Cortex* 25 2752–2762. 10.1093/cercor/bhu074 24735671 PMC6276921

[B13] Frühholz S. Sander D. Grandjean D. (2014b). Functional neuroimaging of human vocalizations and affective speech. *Behav. Brain Sci.* 37 554–604. 10.1017/S0140525X13004020 25514944

[B14] Frühholz S. Trost W. Kotz S. A. (2016). The sound of emotions-Towards a unifying neural network perspective of affective sound processing. *Neurosci. Biobehav. Rev.* 68 1–15. 10.1016/j.neubiorev.2016.05.002 27189782

[B15] Gruters K. G. Groh J. M. (2012). Sounds and beyond: multisensory and other non-auditory signals in the inferior colliculus. *Front. Neural Circ.* 6:96 10.3389/fncir.2012.00096 PMC3518932 23248584

[B16] Jürgens U. (2002). Neural pathways underlying vocal control. *Neurosci. Biobehav. Rev.* 26 235–258. 10.1016/S0149-7634(01)00068-69 11856561

[B17] Klaas H. S. Fruhholz S. Grandjean D. (2015). Aggressive vocal expressions—an investigation of their underlying neural network. *Front. Behav. Neurosci.* 9:121 10.3389/fnbeh.2015.00121 PMC4426728 26029069

[B18] Kleber B. Veit R. Birbaumer N. Gruzelier J. Lotze M. (2010). The brain of opera singers: experience-dependent changes in functional activation. *Cereb. Cortex* 20 1144–1152. 10.1093/cercor/bhp177 19692631

[B19] Kleber B. Zeitouni A. G. Friberg A. Zatorre R. J. (2013). Experience-dependent modulation of feedback integration during singing: role of the right anterior insula. *J. Neurosci.* 33 6070–6080. 10.1523/JNEUROSCI.4418-12.2013 23554488 PMC6618920

[B20] Kosslyn S. M. Ganis G. Thompson W. L. (2001). Neural foundations of imagery. *Nat. Rev. Neurosci.* 2 635–642. 10.1038/35090055 11533731

[B21] Kotz S. A. Schwartze M. (2010). Cortical speech processing unplugged: a timely subcortico-cortical framework. *Trends Cogn. Sci.* 14 392–399. 10.1016/j.tics.2010.06.005 20655802

[B22] Kraemer D. J. M. Macrae C. N. Green A. E. Kelley W. M. (2005). Musical imagery: sound of silence activates auditory cortex. *Nature* 434:158. 10.1038/434158a 15758989

[B23] Prat Y. Taub M. Yovel Y. (2015). Vocal learning in a social mammal: demonstrated by isolation and playback experiments in bats. *Sci. Adv.* 1:e1500019. 10.1126/sciadv.1500019 26601149 PMC4643821

[B24] Roswandowitz C. Schelinski S. von Kriegstein K. (2017). Developmental phonagnosia: linking neural mechanisms with the behavioural phenotype. *Neuroimage* 155 97–112. 10.1016/j.neuroimage.2017.02.064 28254454

[B25] Tremblay-Champoux A. Dalla Bella S. Phillips-Silver J. Lebrun M.-A. Peretz I. (2010). Singing proficiency in congenital amusia: imitation helps. *Cogn. Neuropsychol.* 27 463–476. 10.1080/02643294.2011.567258 21864199

[B26] Trost W. Frühholz S. Cochrane T. Cojan Y. Vuilleumier P. (2014). Temporal dynamics of musical emotions examined through intersubject synchrony of brain activity. *Soc. Cogn. Affect. Neurosci.* 10 1705–1721. 10.1093/scan/nsv060 PMC4666110 25994970

[B27] Xu X. Biederman I. Shilowich B. E. Herald S. B. Amir O. Allen N. E. (2015). Developmental phonagnosia: neural correlates and a behavioral marker. *Brain Lang.* 149 106–117. 10.1016/j.bandl.2015.06.007 26197259

[B28] Zarate J. M. (2013). The neural control of singing. *Front. Hum. Neurosci.* 7:237. 10.3389/fnhum.2013.00237 23761746 PMC3669747

[B29] Zarate J. M. Wood S. Zatorre R. J. (2010). Neural networks involved in voluntary and involuntary vocal pitch regulation in experienced singers. *Neuropsychologia* 48 607–618. 10.1016/j.neuropsychologia.2009.10.025 19896958

