# Supplementary material for: Neural Dynamics of Karaoke-Like Voice Imitation in Singing Performance
Source: Front Hum Neurosci. 2020 Apr 28;14:135. doi: 10.3389/fnhum.2020.00135 (PMC7198696; doi:10.3389/fnhum.2020.00135)
Supplement: Supplementary file 1 [file Data_Sheet_1.PDF]

Supplementary Material

Titel: Neural dynamics of karaoke-like voice imitation in singing performance
Authors: Sascha Frühholz, Wiebke Trost, Irina Constantinescu, and Didier Grandjean

---

Supplemental Tables

Table S1 List of the famous and well-known songs used in this study. From each song we extracted two different 8s excerpts.

Singer	Song title
Vincent Delerm	Fanny Ardant et moi
Louis Armstrong	Hello, Dolly
Jacques Brel	La chanson des vieux
Jacques Brel	La valse à mille temps
Jean-Jacques Goldman	Pas toi
Jean-Jacques Goldman	Puisque tu pars
Mylène Farmer	Rêver
Ray Charles	You Don't Know Me

Table S2 Functional activations for the no-feedback versus the feedback condition during singing voice imitation (experiment 1). **(A)** Higher activity for M.G. compared to the control group, and **(B)** higher activity for the control group compared to M.G.

Region	Cluster size	z value	MNI coordinates		
			x	y	z
**(A) M.G. > CG for no-feedback > feedback**					
R angular gyrus	139	5.89	36	-64	62
L supramarginal gyrus	129	5.36	-48	-44	58
L intraparietal sulcus	242	4.99	-28	-74	54
L superior temporal gyrus	109	4.44	-66	-26	14
L transverse temporal gyrus	674	4.97	-48	-6	-4
L superior temporal gyrus		4.23	-48	14	-18
		3.99	-56	-4	0
		3.9	-62	2	0
L inferior frontal gyrus		4.40	-42	34	-16
		4.01	-56	28	-6
R superior temporal gyrus	70	4.59	56	-54	38
R caudate nucleus	89	4.53	18	22	-2
	330	5.03	6	2	8
		3.56	24	18	-8
R middle frontal gyrus	119	4.33	40	6	60
L precuneus	88	4.27	-2	56	42
	250	3.90	-2	-74	44
R precuneus	187	3.96	2	-50	46
**(B) CG > M.G. for no-feedback > feedback**					
L postcentral gyrus	300	3.97	-42	-30	24
L superior temporal gyrus		3.91	-52	-20	26
L planum temporale		3.35	-40	-42	26
R middle temporal gyrus	513	5.77	52	-68	10
		2.96	40	-60	8
L inferior frontal gyrus	312	5.30	-56	18	22
R inferior frontal gyrus	121	4.41	62	12	22
R caudate nucleus	461	4.52	8	20	10

Table S3 Functional activations for the no-feedback versus the feedback condition during the vocal humming experiment (experiment 2). **(A)** Higher activity for M.G. compared to the control group, and **(B)** higher activity for the control group compared to M.G..

			MNI coordinates		
Region	Cluster size	z value	x	y	z
**(A) M.G. > CG for no-feedback > feedback**					
L medial frontal gyrus	97	4.54	-14	50	20
L superior frontal gyrus (premotor)	59	3.59	-18	6	50
**(B) CG > M.G. for no-feedback > feedback**					
L middle temporal gyrus	67	3.34	-42	-44	0
L precentral gyrus	336	6.10	-62	-4	28
	1787	4.36	-14	46	6
R precentral gyrus		5.74	64	-6	28
L inferior frontal gyrus		3.98	-12	60	0
R inferior frontal gyrus		3.89	62	10	22
R insula	96	4.14	36	-2	14
R inferior occipital gyrus	110	3.54	26	-52	4
L inferior occipital gyrus	464	4.30	-16	-74	4
L cuneus		2.77	-24	-58	2
R cuneus	200	3.94	16	-74	6
L hippocampus	80	3.45	-28	-46	0
R hippocampus		3.16	24	-42	10
L intraparietal sulcus	87	3.22	-28	-74	24

Table S4 Functional connectivity for the no-feedback versus the feedback condition during the singing voice imitation experiment (experiment 1) for several seed regions. Peak activations are reported that showed higher activity for M.G. compared to the control group, or vice versa.

			MNI coordinates		
Region	Cluster size	z value	x	y	z
**Seed region: left aIFG**					
M.G. > CG for no-feedback > feedback					
R caudate nucleus	334	4.22	16	18	20
R planum polare (AC)	92	4.09	46	20	-20
L superior frontal gyrus (anterior)	299	4.03	-28	32	42
R superior frontal gyrus	265	3.98	12	6	38
L posterior cingulate gyrus	61	3.74	-8	-32	30
L caudate nucleus	67	3.5	-10	14	0
CG > M.G. for no-feedback > feedback					
--					
**Seed region: left pIFG**					
M.G. > CG for no-feedback > feedback					
R postcentral gyrus	83	4.06	2	-40	72
L postcentral gyrus	66	3.65	-14	-32	72
CG > M.G. for no-feedback > feedback					
R superior temporal gyrus (mid)	258	4.55	60	10	0
R transverse temporal gyrus (AC)		3.98	64	-6	12
R precentral gyrus	82	4.55	60	0	38
L thalamus	137	4.36	-6	-30	12
R intraparietal sulcus	82	4.23	8	-56	64
R postcentral gyrus	153	4.21	16	-38	72
R superior temporal gyrus (posterior)	57	4.17	64	-40	28
R postcentral gyrus	76	4.14	62	-24	32
R precentral gyrus	88	4.06	20	-22	68
L postcentral gyrus	105	3.97	-22	-36	66
R parietal operculum	65	3.56	40	-20	22
R posterior cingulate gyrus	75	3.44	6	-16	32
**Seed region: right pIFG**					
M.G. > CG for no-feedback > feedback					
--					
CG > M.G. for no-feedback > feedback					
L transverse temporal gyrus (AC)	448	4.47	-50	-8	0
L thalamus	58	4.38	-4	-16	18
L middle frontal gyrus	2020	4.32	-40	56	10
L precentral gyrus		4.24	-36	-10	36
R superior temporal gyrus (posterior)	81	3.73	64	0	-2
R middle frontal gyrus	89	3.44	36	42	12
**Seed region: left SoC**					

M.G. > CG for no-feedback > feedback					
L middle frontal gyrus	126	3.88	-38	26	34
CG > M.G. for no-feedback > feedback					
L precentral gyrus	60	4.09	-36	-16	52
R postcentral gyrus	64	3.96	18	-36	70
L fusiform gyrus	132	3.81	-24	-46	-18
L precuneus	80	3.49	-8	-60	-14
R cereblIum		2.76	0	-48	-14
R insula	60	3.28	36	-16	22
R precentral gyrus		2.75	42	-10	24
**Seed region: right Cd [6 2 8]**					
M.G. > CG for no-feedback > feedback					
L middle frontal gyrus	107	4.03	-34	8	46
R postcentral gyrus	146	3.86	26	-34	58
R superior temporal sulcus (posterior)	112	3.58	58	-36	16
CG > M.G. for no-feedback > feedback					
--					
**Seed region: right Cd [8 20 10]**					
M.G. > CG for no-feedback > feedback					
--					
CG > M.G. for no-feedback > feedback					
R caudate nucleus	115	3.61	18	-28	26
L caudate nucleus	101	3.2	-14	-36	20
**Seed region: left IPS**					
M.G. > CG for no-feedback > feedback					
R intraparietal sulcus	1000	4.77	18	-74	44
L superior frontal gyrus	92	4.05	-6	28	50
R superior frontal gyrus	131	3.94	20	32	48
R precentral gyrus	183	3.94	26	-20	52
L precuneus	176	3.64	-6	-62	40
L intraparietal sulcus		3.21	-16	-72	36
R superior frontal gyrus	85	3.63	8	54	20
L intraparietal sulcus	227	3.55	-28	-78	42
CG > M.G. for no-feedback > feedback					
L planum polare (AC)	122	4.1	-46	-2	-4
L precentral gyrus	211	4.03	-44	-6	36
L thalamus	74	3.78	-6	-28	14
L postcentral gyrus	86	3.71	-44	-22	42
**Seed region: left aSTG**					
M.G. > CG for no-feedback > feedback					
L superior temporal sulcus (posterior)	73	5.57	-46	-54	30
L thalamus	56	5.27	-2	-18	18

L postcentral gyrus	76	4.89	-2	-38	64
R planum polare (AC)	185	4.6	48	18	-20
L inferior temporal gyrus	59	4.46	-50	-52	-12
L precentral gyrus	242	4.45	-56	4	6
L superior temporal gyrus (mid)		3.42	-62	2	0
L superior temporal gyrus (posterior)		3.29	-52	-4	0
L caudate nucleus	85	4.39	-14	12	28
R superior frontal gyrus	64	4.28	26	60	18
R inferior colluculi	91	4.25	10	-32	-8
R thalamus		4.11	16	-32	0
R superior frontal gyrus	175	4.03	12	8	36
R caudate nucleus	345	4.02	22	22	12
R middle frontal gyrus		3.98	48	40	20
R inferior frontal gyrus (anterior)		3.86	30	26	20
L superior frontal gyrus	77	3.88	-2	56	28
R superior temporal gyrus (mid)	78	3.75	62	6	0
R superior temporal gyrus (posterior)		2.89	66	-8	-2
L posterior cingulate gyrus	85	3.74	-8	-34	30
L superior frontal gyrus	114	3.66	-6	6	56
L caudate nucleus	198	3.66	-14	12	4
L precentral gyrus	116	3.65	-50	0	28
L superior frontal gyrus	161	3.62	-20	60	14
L middle frontal gyrus	231	3.52	-20	26	40
R superior temporal gyrus (posterior)	102	3.37	58	-46	26
CG > M.G. for no-feedback > feedback					
--					
**Seed region: left pSTG**					
M.G. > CG for no-feedback > feedback					
L superior temporal gyrus (posterior)	109	4.99	-44	-54	24
R superior frontal gyrus	149	4	20	42	32
R posterior cingulate gyrus	245	3.82	16	-44	32
R intraparietal sulcus		3.57	28	-46	30
R intraparietal sulcus	133	3.74	22	-58	50
R postcentral gyrus	184	3.47	14	-38	56
R postcentral gyrus	78	3.46	32	-34	64
CG > PS for no-feedback > feedback					
L posterior cingulate gyrus	83	3.29	-2	-48	-8

Table S5 Functional connectivity for the no-feedback versus the feedback condition during the vocal humming experiment (experiment 2) for several seed regions. Peak activations are reported that showed higher activity for M.G. compared to the control group, or vice versa.

			MNI coordinates		
Region	Cluster size	z value	x	y	z
**Seed region: left MFC [-14 50 20]**					
M.G. > CG for no-feedback > feedback					
R amygdala	133	4.53	24	-6	-18
R hippocampus		3.11	14	-16	-16
R thalamus	132	3.7	8	-8	0
L amygdala	482	3.65	-22	-8	-12
L frontal operculum		3.49	-38	-4	6
L planum polare (AC)		3.45	-34	-2	-18
L inferior frontal gyrus	135	3.37	-32	34	8
CG > M.G. for no-feedback > feedback					
R middle frontal gyrus	64	3.74	30	18	56
R superior frontal gyrus	92	3.52	10	40	42
L angular gyrus	80	3.42	-34	-76	28
R precentral gyrus	61	3.17	18	-14	74
**Seed region: left MFC [-12 60 0]**					
M.G. > CG for no-feedback > feedback					
R superior frontal gyrus	74	3.23	18	36	44
CG > M.G. for no-feedback > feedback					
R amygdala	70	4.11	26	-6	-18
R hippocampus		2.74	30	-16	-12
R insula	181	3.59	34	12	0
R inferior frontal gyrus (posterior)		3.09	42	16	6
L insula	88	3.27	-38	-4	6
R cuneus	81	3.25	8	-78	0
L middle frontal gyrus	57	2.96	-26	36	12
**Seed region: right IFG**					
M.G. > CG for no-feedback > feedback					
R superior frontal gyrus	725	4.33	4	62	32
R precentral gyrus	79	4.18	18	-12	72
L postcentral gyrus	345	4.1	-24	-28	56
R postcentral gyrus	87	3.93	36	-26	66
R superior frontal gyrus	65	3.62	4	58	18
CG > M.G. for no-feedback > feedback					
R thalamus	74	3.7	2	-14	-2
**Seed region: right aINS**					
M.G. > CG for no-feedback > feedback					
L precentral gyrus	169	5.14	-24	-26	56
L postcentral gyrus	70	4.23	-34	-24	68

L supramarginal gyrus	83	4.04	-52	-34	56
R occipital gyrus	70	4.03	2	-64	-2
L occipital gyrus	58	3.84	-16	-50	10
L middle frontal gyrus	124	3.56	-60	22	24
L inferior frontal gyrus (posterior)		3.47	-64	8	24
R middle frontal gyrus	125	3.47	28	40	16
L cuneus	61	3.46	-16	-80	12
R cuneus	75	3.32	22	-72	12
CG > M.G. for no-feedback > feedback					
R thalamus	68	4.31	0	-16	-4
L postcentral gyrus	133	3.88	-12	-28	82
R intraparietal sulcus	114	3.38	20	-54	76
**Seed region: left MC**					
M.G. > CG for no-feedback > feedback					
L cuneus	190	3.84	-16	-68	14
R intraparietal sulcus	61	3.19	24	-66	34
CG > M.G. for no-feedback > feedback					
L superior frontal gyrus	151	3.91	-26	8	60
**Seed region: right MC**					
M.G. > CG for no-feedback > feedback					
R superior frontal gyrus	3408	6.34	4	64	32
L intraparietal sulcus	1306	4.48	-20	-76	38
L precentral gyrus	112	4.04	-24	-16	62
L occipital gyrus	87	3.99	-14	-90	14
L postcentral gyrus	66	3.73	-26	-30	58
R intraparietal sulcus	120	3.67	32	-42	64
CG > M.G. for no-feedback > feedback					
R thalamus	7211	6.42	2	-14	-4
R amygdala		6.33	28	0	-20
R lingual gyrus	170	4.78	20	-54	-4
R precuneus	316	4.29	20	-38	34
L parahippocampal gyrus		3.21	-22	-36	-12
L middle temporal gyrus	56	3.8	-48	14	-20
R caudate nucleus	167	3.72	10	16	8
L caudate nucleus	89	3.43	-12	16	6
**Seed region: left IPS**					
M.G. > CG for no-feedback > feedback					
L postcentral gyrus	384	4.97	-34	-24	70
R precentral gyrus	662	4.46	18	-12	72
R superior frontal gyrus	63	4.07	16	50	38
R precentral gyrus	64	4.01	32	6	26
L supramarginal gyrus	63	4.01	-50	-34	54
L intraparietal sulcus		3.13	-46	-44	58

R middle frontal gyrus	236	3.98	24	32	34
R cuneus	76	3.78	26	-72	10
L middle frontal gyrus	118	3.71	-28	18	42
L inferior frontal gyrus (posterior)	74	3.37	-58	12	24
CG > M.G. for no-feedback > feedback					
--					
